# Pattern-based mining strategy to detect multi-locus association and gene × environment interaction

**DOI:** 10.1186/1753-6561-1-s1-s16

**Published:** 2007-12-18

**Authors:** Zhong Li, Tian Zheng, Andrea Califano, Aris Floratos

**Affiliations:** 1 Department of Computational Genetics, High Throughput Biology Inc., 513 West Mount Pleasant Avenue, Livingston, New Jersey 07039, USA; 2 Department of Statistics, Columbia University, Room 1005, MC4690, 1255 Amsterdam Avenue, New York, New York 10027, USA; 3 Department of Biomedical Informatics, Columbia University, 622 West 168th Street, Vanderbilt Clinic, 5th Floor, New York, New York 10032, USA; 4 Center for Computational Biology and Bioinformatics, Columbia University, 1130 St. Nicholas Avenue, New York, New York 10032, USA; 5 Co-senior author

## Abstract

As genome-wide association studies grow in popularity for the identification of genetic factors for common and rare diseases, analytical methods to comb through large numbers of genetic variants efficiently to identify disease association are increasingly in demand. We have developed a pattern-based data-mining approach to discover unlinked multilocus genetic effects for complex disease and to detect genotype × phenotype/genotype × environment interactions. On a densely mapped chromosome 18 data set for rheumatoid arthritis that was made available by Genetic Analysis Workshop 15, this method detected two potential two-locus associations as well as a putative two-locus gene × gender interaction.

## Background

Long considered to hold promise for dissecting the genetic etiology of complex diseases [1], genome-wide association studies have produced significant and sometimes highly reproducible findings in an increasing number of publications [2]. Despite early successes that demonstrated the feasibility of whole-genome studies, strategies for fully analyzing genome-wide data, such as detecting multilocus genetic effects for complex disease or detecting genotype × phenotype/genotype × environment interactions, are still underdeveloped or underutilized, affecting the effectiveness of those large-scale studies on advancing our understanding of the genetic contribution to common human diseases [3,4]. An increasing number of methods have been proposed to detect multilocus genetic association: some make use of haplotypes [5] or logistic regression [6], while others use nonparametric "data mining" strategies such as the multifactor dimensionality reduction (MDR) [7] and neural networks [8]. Methods to analyze pair-wise interactions between unlinked loci have also been developed [9].

We have developed a pattern-based mining strategy (manuscript in preparation) to detect local (markers in moderate or strong linkage disequilibrium, or LD) and global (unlinked markers) multilocus genetic associations as well as gene × gene/gene × environment interactions. The pattern-based method exhaustively yet efficiently identifies all patterns satisfying pre-defined pattern search criteria and evaluates the association of patterns to disease state through a χ^2^-based test statistics. For the work described in this report, we applied the pattern-based method on the chromosome 18 data set on rheumatoid arthritis (RA) ascertained by the North American Rheumatoid Arthritis Consortium (NARAC) that was made available for the Genetic Analysis Workshop 15 (GAW15). Ten patterns were found to be significantly associated with the RA phenotype after multiple testing correction. The significance of those ten patterns was confirmed using Monte Carlo simulation. Furthermore, we identified a potentially significant multi-gene/gender interaction involving two loci: SNP0177 and the LD region containing markers SNP0603–SNP0615.

## Methods

### Pattern Examiner: a pattern-based method to detect multi-locus association and interaction

Data are organized in a two-dimensional data matrix with markers as columns, individuals as rows, and individuals' alleles or genotypes as cell values. Each marker is represented by five columns: one column for each of the two alleles of the marker and one column for each of the three possible genotypes for the marker. A pattern is defined as a maximal sub-matrix of the data matrix in which the value of each marker across all individuals in the sub-matrix satisfies a predefined equivalence criterion such as same genotype value. A sub-matrix is maximal if 1) no more rows can be added while keeping the columns fixed and, 2) no more columns can be added when keeping the rows fixed. Under this formulation patterns can be used to model both multilocus allelic and multilocus genotypic contributions to disease state.

Pattern Examiner is a nonparametric data mining-based method for the detection of multilocus associations and gene × gene/gene × environment interactions on data collected in population-based case/control studies. This method has two steps: 1) pattern discovery and 2) significance evaluation. In the pattern discovery step, patterns are identified using as input data from the case population only. The extensiveness (and execution time) of the pattern discovery step is controlled by two parameters: the *support threshold*, which specifies the minimum number of rows a pattern must have; and the *locus threshold*, which specifies the extent of locus interaction. For example, with the support and locus thresholds set to 20 and 2 respectively, all reported patterns will have 20 or more case supports and mostly one or two markers. In the significance evaluation step, a 2 × 2 contingency table is constructed for each pattern to tally its support in the case and control populations ("case support" and "control support", respectively). The two categorical variables tabulated are population type ("cases" vs. "controls") and pattern match status ("matches" vs. "does not match"). Partially missing data are excluded. The *p*-value is obtained from a χ^2 ^test of independence and then adjusted for multiple testing. A modified Bonferroni correction for multiple testing is applied to each pattern, using as the correction factor the total number of patterns that contain equal or greater case support than the target pattern under significance evaluation, rather than the total number of patterns identified. As a result, the adjusted significance is robust against the arbitrary selection of values for parameters in the pattern discovery step. The odds ratio with confidence interval is also calculated for each pattern.

### Test for differential gene × gender interactions in cases vs. controls

To test for the null hypothesis that there is no difference on genotype × gender interaction between cases and controls, three 2 × *N *contingency tables were constructed with the observed *N *genotypes of a significant pattern as rows and genders as columns. Three chi-square values, χ^2^_case_, χ^2^_control_, and χ^2^_pooled_, were then obtained for cases only, controls only, and cases and controls pooled, respectively. The *p*-value was obtained using χ^2 ^= χ^2^_case _+ χ^2^_control _- χ^2^_pooled _with *N *- 1 degree of freedom.

## Results

### Significant association between two-locus patterns and RA

We identified 2.6 million patterns, mostly containing two markers, from the NARAC chromosome 18 data set, for a support threshold of 20 and a locus threshold of 2. From this set, 65,689 patterns were found to have *p*-values ≤ 0.01 before multiple testing correction. After Bonferroni correction, ten patterns remained significant, as shown in Table 1 (Column "Adjusted *p*-value"). Interestingly, all significant patterns share the following characteristics: 1) they all contain marker SNP0177 with allele 1, suggesting a dominant effect for marker SNP0177; 2) they all have odds ratio around two, suggesting a modest relative risk; 3) they all have large number of case support (more than 50% of all cases), suggesting a rather common inheritance; and 4) they are all mapped to intergenic regions on chromosome 18. Furthermore, except for markers SNP672 and SNP0177, all other markers in those ten patterns are mapped to two LD blocks as identified by the HapBlock program [10] (markers SNP1130 and SNP 1131; markers SNP0603, SNP0604, SNP0605, SNP0606, SNP0608, SNP0609, SNP0610, and SNP0615). Similar to marker SNP0177, single alleles for markers 1130 and 1131 were found to contribute to the significant patterns, suggesting a dominant effect. On the other hand, all markers in the SNP0603–0615 LD block consistently demonstrated a recessive effect with the inclusion of homozygote genotype(s) in corresponding significant patterns.

**Table 1:** Significant patterns identified with the pattern-based method

Markers in pattern	No. of cases (F/M ratio)^a^	No. of controls (F/M ratio)	Odds ratio (confidence interval)	Unadjusted *p*-value^b^	Adjusted *p*-value^c^
SNP0177 (allele 1); SNP1131 (allele 1)	260 (3.9)	178 (3.3)	2.06 (1.58–2.68)	5.04 × 10^-8^	0.00185
SNP0177 (allele 1); SNP0610 (genotype 2/2)	259 (3.7)	180 (3.5)	2.0 (1.54–2.61)	1.57 × 10^-7^	0.00601
SNP0177 (allele 1); SNP1130 (allele 2)	234 (4.3)	154 (3.6)	2.06 (1.58–2.69)	8.03 × 10^-8^	0.00663
SNP0177 (allele 1); SNP0615 (genotype 1/1)	259 (3.7)	182 (3.4)	1.97 (1.51–2.55)	3.08 × 10^-7^	0.01178
SNP0177 (allele 1); SNP0603 (genotype 1/1)	229 (3.7)	151 (4.2)	2.02 (1.55–2.65)	1.49 × 10^-7^	0.01396
SNP0177 (allele 1); SNP0604 (genotype 2/2); SNP0605 (genotype 2/2)	258 (3.8)	182 (3.4)	1.95 (1.5–2.54)	4.42 × 10^-7^	0.01763
SNP0177 (allele 1); SNP0609 (genotype 1/1)	234 (3.6)	158 (3.7)	1.98 (1.52–2.58)	3.41 × 10^-7^	0.02822
SNP0177 (allele 1); SNP0606 (genotype 2/2)	255 (3.9)	181 (3.4)	1.92 (1.48–2.49)	8.68 × 10^-7^	0.03879
SNP0177 (allele 1); SNP0672 (allele 2)	273 (3.5)	202 (3.5)	1.86 (1.43–2.42)	2.33 × 10^-6^	0.04072
SNP0177 (allele 1); SNP0608 (genotype 1/1)	233 (3.6)	158 (3.7)	1.96 (1.5–2.56)	4.91 × 10^-7^	0.04161

Significant patterns were ordered according to their adjusted *p*-values. Each pattern contains markers with either individual allele or genotype.

^a^The "No. of cases" column contains the number of individuals with RA who carried the specified alleles/genotypes at the specified markers in a pattern. The number in parenthesis indicates the female/male ratio. Similar arrangement was made for "No. in controls".

^b^*p*-Value before the Bonferroni correction.

^c^*p*-Value obtained after the Bonferroni correction.

To further evaluate the significance of those ten patterns, we performed a Monte Carlo simulation using 400 simulated data sets generated by randomizing the case-control assignment of the 920 individuals in the study while maintaining the female/male ratio in both cases and controls. The pattern-based method was applied to each simulated data set. On average, 2.7 million patterns were identified from each data set, which were then subject to test statistics and multiple testing correction. In 8 out of 400 (2%) simulated data sets we observed ten or more patterns that had *p*-values less than 0.05 after multiple testing correction (false-positive patterns). In 20 out of 400 (5%) simulated data sets we observed five or more false-positive patterns. These results suggest that the ten significant patterns discovered in the real data set are unlikely to be an artifact of chance alone. Furthermore, if we consider the fact that these ten patterns share the same marker, SNP0177, then the significance gets even stronger: out of the 400 simulated data sets there was only 1 case (0.25%) where sharing of a common marker was observed in ten or more patterns.

### Significant interaction between multilocus genotypes and gender in RA

It is known that the incidence rate of RA is higher in females. Indeed, there is a matching 3.82:1 female/male ratio in both the case sample and control sample of this data set. However, as shown in Table 1 (columns "No. of cases (F/M ratio)" and "No. of controls (F/M ratio)"), notable differences on the female/male ratio between cases and controls were observed for many significant patterns. To investigate the genotype × gender interaction further, we constructed 2 × *N *contingency tables for each marker in the significant patterns and for each significant pattern with observed genotypes for marker or pattern as rows. Four variations of the contingency tables were constructed as detailed in the legend of Table 2. Table 2 shows a representative pattern demonstrating significant interaction between multilocus genotypes and gender. Individually, marker SNP0177 or SNP0615 was not significant in male population (bold). However together they demonstrated strong significance in male population that reached the same magnitude (*p *= 0.00118) as in females (*p *= 0.00109) despite the much smaller sample size. When both female and male populations were considered together ("With gender partition"), an even stronger association between the pair of markers and the disease was observed. For individuals with the 1/1–1/2 genotype for markers SNP0177 and SNP0615, an odds ratio of 11.7 (confidence interval: 1.34–102.86) was observed in females over males. For individuals with the 2/2–1/2 genotype for markers SNP0177 and SNP0615, an odds ratio of 7.51 (confidence interval: 1.94–28.99) was observed in males over females. Logistic regression analysis on the interaction between the pattern and gender also yielded a significant *p*-value of 0.0021 (data not shown). Furthermore, a test for differential interaction in cases and controls yielded a *p*-value of 0.0016, suggesting that there is significant interaction between the SNP0177–SNP0615 pattern and gender in the affected individuals vs. unaffected individuals. Similar results were obtained for all patterns containing markers in the SNP0603–0615 LD block (data not shown). On the other hand, no differential interaction in cases and controls was observed for the three remaining significant patterns (data not shown).

**Table 2:** Evidence of interaction between multiple loci and gender in cases

Marker Names	Type^a^	*p*-Value
SNP0177	Female only	*0.006738*
SNP0177	Male only	**0.105399^b^**
SNP0177	With gender partition	0.012727
SNP0177	Without gender partition	0.013569
		
SNP0615	Female only	0.002029
SNP0615	Male only	**0.246597**
SNP0615	With gender partition	0.009541
SNP0615	Without gender partition	0.004517
		
SNP0177–SNP0615	Female only	0.001092
SNP0177–SNP0615	Male only	**0.001182**
SNP0177–SNP0615	With gender partition	0.000024
SNP0177–SNP0615	Without gender partition	0.000862

^a^Four variations of the 2 × *N *contingency table were constructed for each marker in the significant patterns in Table 1 and for each significant pattern with the observed *N *genotypes as rows. Each contingency table has two columns for case and control. In "Without gender partition", individuals were grouped and allocated to table cells according to genotypes regardless of their gender. In "With gender partition", individuals were grouped together according to genotypes and gender with each combination of genotype and gender as a row. In "Males only", only males were grouped and allocated to table cells according to genotypes. In "Females only", only females were grouped and allocated to table cells according to genotypes.

^b^Bold font indicates results for males only.

## Discussion

Several lines of evidence suggest that the significant multilocus associations we detected with Pattern Examiner on the NARAC chromosome 18 data set provided by GAW15 might be true associations: 1) except for marker SNP0177 and SNP0672, multiple markers from the same LD region (markers SNP1130 and 1131, markers SNP0603–0615) were found to be in different significant patterns; 2) all markers in the same LD region displayed consistent dominant (an allele in the pattern) or recessive (a homozygote genotype in a pattern) effect; 3) a Monte Carlo simulation with randomized cases and controls produced a type I error probability of 0.02 for the observed results. The singleton SNP0177 does raise a flag because it is in a strong LD region with adjacent markers. Further investigation is necessary to confirm the role SNP0177 plays in the association. The ultimate proof for true association will have to come from replication studies. Taking advantage of our method's ability to detect multilocus association, we performed a novel multilocus gene × gender interaction analysis and detected a two-locus gene × gender interaction that was supported by three independent assays. Detailed analysis revealed large odds ratios for certain genotype combinations. However, a wide confidence interval (due to small sample size) dampens our enthusiasm. Again, further investigation with larger sample sizes is necessary to confirm this observation.

Several reports in the "Association – Problem 2" group of GAW15 have identified the LD region including markers SNP1097–1107 to be significantly associated with RA [[11]; Zhu G, Rao S, Li X, personal communication]. Although we also found markers SNP1097–1107 to be significant using a genotype-based single-marker χ^2 ^test (*p *= 0.0002 for most markers in this region), the significance disappeared after Bonferroni correction with 2300 markers. After applying a less stringent Bonferroni correction using the number of LD blocks (233 LD blocks were identified by the HapBlock program) in this 10-Mb region, those markers resurfaced as significant. Because none of the significant patterns identified by the pattern-based method included markers SNP1097–1107, this locus might act alone as a risk factor for RA.

By focusing on detecting multilocus association and interactions, the pattern-based method complements the traditional single marker association test. A preliminary power analysis (data not shown) suggested that this method has more power than the traditional single marker-based analysis under a couple of two-locus disease models. Additional power analysis is needed to further validate its utility.

## Conclusion

We have identified two potential multilocus associations (SNP0177/SNP1130–1131 and SNP0177/SNP0603–0615) with RA as well as evidence of interaction between gender and loci SNP0177/SNP0603–0615.

## Competing interests

Dr. Zhong Li is currently employed by High Throughput Biology Inc. (HTB) and serves as its President. He owns stocks of HTB and has the potential to gain commercially from this publication. No patent was filed at this time on the methods discussed in the manuscript. Dr. Andrea Califano is currently consulting and on the Advisory Board of High Throughput Biology Inc.(HTB), for whichhe receives a very small monthly compensation. He has no stock nor holds any position in any other company that stands to commercially gain from this publication. He has no further conflict of interest. No patent was filed at this time on the methods discussed in the manuscript. Dr. Aris Floratos has received consulting fees from High Throughput Biology Inc. He has no stock nor holds any position in any other company that stands to commercially gain from this publication. He has no further conflict of interest. No patent was filed at this time on the methods discussed in the manuscript. Dr. Tian Zheng has no competing interests.
